# Isosteviol Sodium Protects against Ischemic Stroke by Modulating Microglia/Macrophage Polarization via Disruption of GAS5/miR-146a-5p sponge

**DOI:** 10.1038/s41598-019-48759-0

**Published:** 2019-08-21

**Authors:** Hao Zhang, Minyi Lu, Xiaofeng Zhang, Yihe Kuai, Ying Mei, Qiwen Tan, Kailun Zhong, Xiaoou Sun, Wen Tan

**Affiliations:** Institute of Biomedical and Pharmaceutical Sciences, Guangdong University of Technology, Guangzhou, China

**Keywords:** Neuroimmunology, Neurological disorders

## Abstract

Recent studies have shown that transforming microglia phenotype from pro-inflammation of M1 phenotype to anti-inflammation and tissue-repairing M2 phenotype may be an effective therapeutic strategy for preventing ischemic stroke brain injury. Isosteviol Sodium (STV-Na) has shown promise as a neuroprotective agent in cerebral ischemia model, although its effect on microglial polarization and chronic recovery after stroke is not clear. Here, we demonstrated that STV-Na treatment significantly reduced cerebral ischemic damage at both acute and chronic time points. STV-Na has a profound regulatory effect on microglia response after stroke. It can promote M2 polarization and inhibit microglia-mediated inflammation (M1) response following stroke *in vivo* and *in vitro*. Furthermore, we also found that Growth Arrest-Specific 5 (GAS5) altered OGD/R-induced microglial activation by increasing Notch1 expression via miR-146a-5p, the mRNA level of GAS5 and the protein level of Notch1 *in vivo* and *in vitro*, were discovered that both downgraded with STV-Na. Taken together, the present study demonstrated that STV-Na exerted neuroprotective effects by modulating microglia/macrophage polarization in ischemic stroke via the GAS5/miR-146a-5p sponge. These findings provide new evidence that targeting STV-Na could be a treatment for the prevention of stroke-related brain damage.

## Introduction

Stroke, as a leading “killer” endangering human health, is one of the three major diseases causing the highest lethality and disability rates globally, accompanied by ischemic stroke accounting for approximately 80% of all strokes^1–3^. Currently, most treatments for cerebral ischemia are focused on thrombolysis and neuroprotection, and clinical therapeutic drugs are fairly limited, among which recombinant tissue plasminogen activator (rtPA) is used most widely. However, due to the relatively narrow time window and potential bleeding injury risk, rtPA is rendering its use relatively uncommon^4^. The goal of neuroprotection is to protect cells in the ischemic penumbra region of brain tissue from death^5,6^. Many of the candidate neuroprotective drugs have failed in clinical trials, despite nearly a decade of research and some potential efficacy in animal models^7,8^. Thus, it appears that a focus solely on salvaging the penumbra without repairing the microenvironment in the damaged brain tissue timely is not enough for subsequent neuroprotection and functional recovery after stroke^9^. Therefore, it is critical that new therapeutic strategies maintain a brain balance by actively mediating cell survival and regeneration.

Microglia and infiltrating macrophages are extremely important in regulating the brain’s immune and inflammatory response following ischemic injury^10^. Increasing evidence reveals that microglia/macrophages exhibit different phenotypes and functions in the ischemic brain injury process^11,12^. Microglia are immune cells of the central nervous system that act as sensors in normal brain. Although microglia may protect the brain in some cases, uncontrollable inflammation overestimates its beneficial effects to a large extent. It is well known that microglia are similar to peripheral macrophages and can respond to micro-environmental interference by altering phenotype and function. Classical activation phenotype (M1) and alternative activation phenotype (M2) are two recognized phenotypes. Although similar taxonomic systems have been used, many differences have been found between microglia and peripheral macrophages. Therefore, some studies have shown that this differentiation is still controversial, and emphasized the need to differentiate microglia based on transcriptional and proteomic analysis, taking into account their stimulation and disease specificity^13–15^.

Several researches have also shown that microglia may be stationary or active. This debate has required nearly two years of research to establish that microglia are involved in intracranial sentinel function, and for which there is no such thing as a resting state^16,17^. In addition, many studies have shown that the M2 population has neuroprotective effects after stroke^18,19^. The M2 phenotype plays a protective role in the brain by promoting the ability of brain tissue repair, debris phagocytosis and regeneration after cerebral ischemia. However, inflammatory cytokines induced by M1 phenotype aggravate brain injury after stroke. Therefore, the status of microglia after cerebral ischemia is very important for the prognosis^18^. For example, alternatively activated M2 microglia phenotype inhibits inflammation and promotes tissue repair by producing neurotrophic and anti-inflammatory factors^20^. However, classically activated M1 microglia tend to release pro-inflammatory cytokines that aggravate tissue injury^18^, although they are also involved in removing cell debris both acutely and chronically following stroke. This phenotypic plasticity and diversity indicate that microglia are important and unique resident macrophages, and play an important role in the immune defense against ischemic injury^12^. Therefore, regulating the polarization of microglia to M2 phenotype may be essential to alleviate inflammatory damage and microglia activation following stroke^20^.

Long noncoding RNAs (lncRNA > 200 nucleotides in length) are a non-protein-coded transcript that play an important role in a wide variety of physiological and pathological processes^21^. The processes regulated by lncRNAs include apoptosis, DNA damage, recruitment of histone modification complexes and co-activation of neighboring genes^22,23^. Thus far, a large number of literatures have confirmed that a considerable number of lncRNAs are involved in the pathogenesis of ischemic stroke^24,25^, with mutations and lncRNA expression in various forms of brain dysfunctions pointing towards their potential roles in brain physiology and pathology. Among these lncRNAs, growth arrest specific 5 (GAS5) was named on the basis that its increased expression was associated with rapamycin induced cell cycle arrest, which was first detected in mouse fibroblasts isolated from NIH 3T3^26^. Previous research has confirmed that GAS5 regulates cell viability negatively and is up-regulated in the neuronal hypoxia response. Recent work has also revealed that GAS5 which acts as a competing endogenous RNA regulate neuronal coding genes in the contexts of methamphetamine-induced neuronal apoptosis^27^, microglial M2 polarization, and exacerbated demyelination^28^.

Isosteviol sodium (STV-Na), is a sesquiterpene diterpenoid obtained by hydrolysis of stevioside acid^29^. Multiple studies have demonstrated that STV-Na has neuroprotective effects in experimental models^30,31^. For instance, treatment with STV-Na was demonstrated to effectively reduce the size of infarction and improve the neurobehavioral function outcomes^32,33^. Several mechanisms likely underlie the therapeutic effects of STV-Na, such as anti-oxidative, reduced inflammatory response, and anti-apoptotic mechanisms^30^, as well as the inhibition of mitochondrial fission^34^. Despite this, however, the effects and mechanisms of STV-Na in phenotypic polarization of microglia after ischemic stroke remain to be explored.

In our current study, we evaluated the effect of STV-Na on microglial polarization and inflammatory response both *in vitro* and *in vivo* simulated ischemic stroke models. The results showed that STV-Na had neuroprotective effects *in vivo* and *in vitro*, promoting M2 polarization of microglia and inhibiting M1 polarization. Previous studies have shown that lncRNAs compete for microRNA response elements to regulate gene expression from epigenetics, thus acting as natural microRNA sponges and reducing the binding of endogenous microRNAs to their target genes. Therefore, we provide evidence that miR-146a-5p molecular sponge GAS5 is destroyed by STV-Na, and that STV-Na is increasingly considered as a potential drug for the treatment of cerebral ischemia. These findings demonstrate that STV-Na prevents ischemic stroke by regulating microglia/macrophage polarization via modulation of GAS5/miR-146a-5p sponge.

## Results

### STV-Na protected against brain damage after MCAO/R

**Figure 1:**
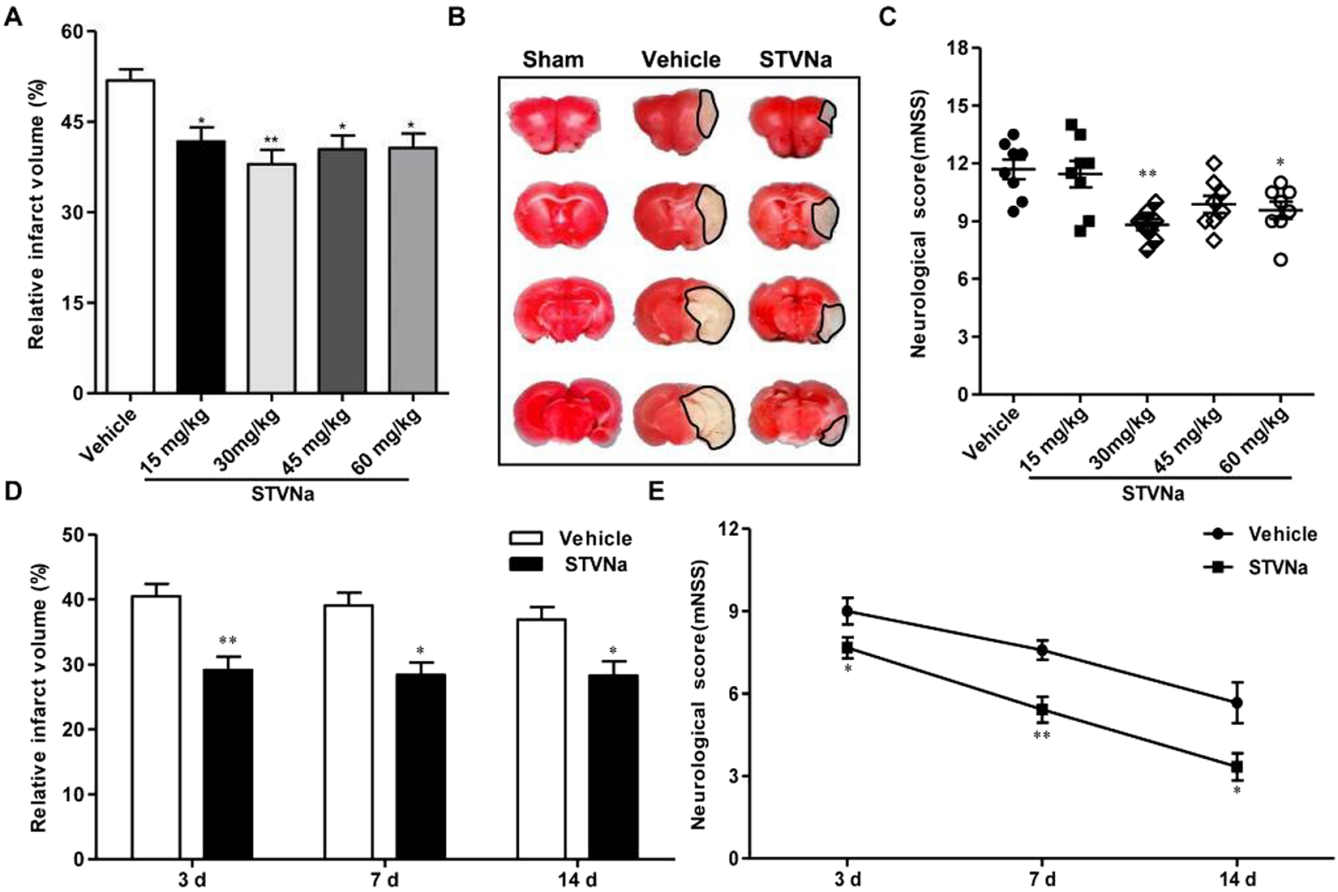
Effect of STV-Na on behavioral deficits and brain infarct volume in mice with MCAO/R injury. Multiple tests were performed in mice that underwent 1 h of ischemia followed by 1 d, 3 d, 7 d or 14 d of reperfusion. (**A**) Dose-dependent effects of STV-Na on infarct volumes evaluated by TTC at 24 h (n = 6 per group). (**B**) A sample of brain slices TTC staining at 24 h after MCAO/R. (**C**) MNSS was evaluated at 24 h (n = 6 per group). (**D**) Effects of STV-Na on infarct volumes at different time points (n = 6 per group). (**E**) MNSS was evaluated at different time points (n = 6 per group). Data are represented as mean ± SD, ^*^P < 0.05, ^**^P < 0.01.

To investigate the effects of STV-Na in a mouse model of transient cerebral focal ischemia, we calculated infarct volume and functional improvements 24 hours after MCAO. As shown in Fig. 1A–C, STV-Na doses of 30 mg/kg significantly reduced infarct size and improved functional outcomes in ischemic animals as compared to vehicle-treated animals. This dose was therefore selected for subsequent experiments. Next, 30 mg/kg STV-Na was administered intraperitoneally immediately after MCAO and reperfusion at 3, 7, and 14 days following surgery. As shown in Fig. 1D,E, STV-Na treatment also had neuroprotective effects at 3, 7 and 14 days post-stroke. These data reveal improvements in some aspects of neurological function and infarct volume at both acute and chronic time points after stroke with STV-Na.

### STV-Na treatment inhibits M1 polarization and promotes M2 polarization of microglia after MCAO/R *in vivo*

**Figure 2:**
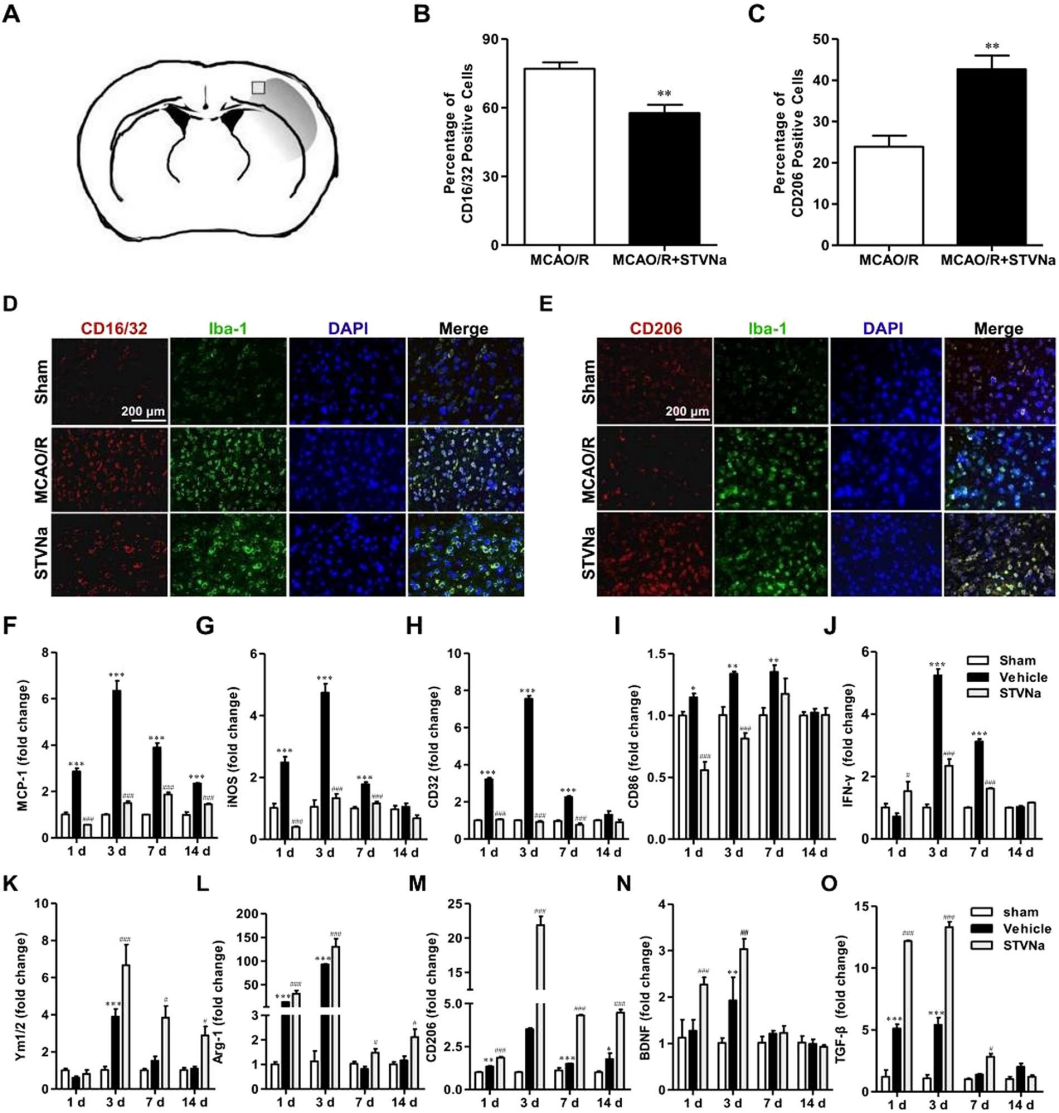
STV-Na treatment enhances M2 polarization and suppresses M1 polarization of microglia in the ischemic cortex after MCAO/R. Representative double-immunofluorescence staining for CD16/32 or CD206 and Iba-1 markers in brain sections obtained from STV-Na or vehicle-treated mice 3 days after MCAO/R, or from sham-operated mice. Scale bar: 200 μm. (**A**) Sketch picture showing the position of the immunofluorescence pictures obtained from the peri-infarct of cortex. (**B**) Quantification of the percentage of CD16/32^+^/Iba-1^+^ cells among total Iba-1^+^ cells. (**C**) Quantification of the percentage of CD206^+^/Iba-1^+^ cell among total Iba-1^+^ cells. (**D**) Cortex sections co-stained for CD16/32 (M1 marker) (red) and Iba-1(green). (**E**) Cortex sections co-stained for CD206 (M2 marker) (red) and Iba-1 (green). (**F**–**O**) RT-PCR analysis of M1 (MCP-1, iNOS, CD32, CD86, IFN-γ) and M2 (Ym1/2, Arg-1, CD206, BDNF, TGF-β) markers in the ischemic cortex at 1, 3, 7, 14 days after MCAO/R. Data are represented as mean ± SD, ^*^P < 0.05, ^**^P < 0.01, ^***^P < 0.01, versus Sham; ^#^*P* < 0.05, ^##^*P* < 0.01, ^###^*P* < 0.001, versus Vehicle group.

Microglia polarization plays a critical role in the pathological progression of ischemic stroke^18^. Given this, cortical penumbra of brain sections were double stained with anti-Iba-1 (microglial marker) and -CD16/32 (M1 marker) or -CD206 antibodies. As shown in Fig. 2B,D, the percent of CD16/32^+^Iba-1^+^ cells (of total Iba-1^+^ microglia/macrophages) was significantly higher in vehicle-treated mice than in STV-Na-treated stroke mice. Moreover, STV-Na administration significantly increased the percent of CD206^+^Iba-1^+^ M2 microglia/macrophages after MCAO (Fig. 2C,E).

To further assess whether STV-Na regulated microglial polarization in the brain after MCAO/R, the effects of STV-Na on microglial polarization were examined via RT-PCR. RNA samples were prepared from MCAO/R mice treated with STV-Na or vehicle. As shown in Fig. 2, the expression of M1 (*MCP-1*, *iNOS*, *CD32*, *CD86* and *IFN-γ*) and M2 (*Ym1/2*, *Arg-1*, *CD206*, *BDNF and TGF-β*) markers was significantly increased in vehicle-treated stroke mice 1, 3, 7, and 14 days after MCAO. STV-Na treatment significantly inhibited this increase in M1 markers expression and decrease in M2 markers expression after MCAO/R (Fig. 2F–O). Taken together, these results demonstrate that STV-Na treatment promoted M2 polarization and inhibited M1 polarization after stroke, consistent with its neuroprotective properties.

### Administration of STV-Na promoted M2 polarization and inhibited M1 polarization after OGD/R *in vitro*

**Figure 3:**
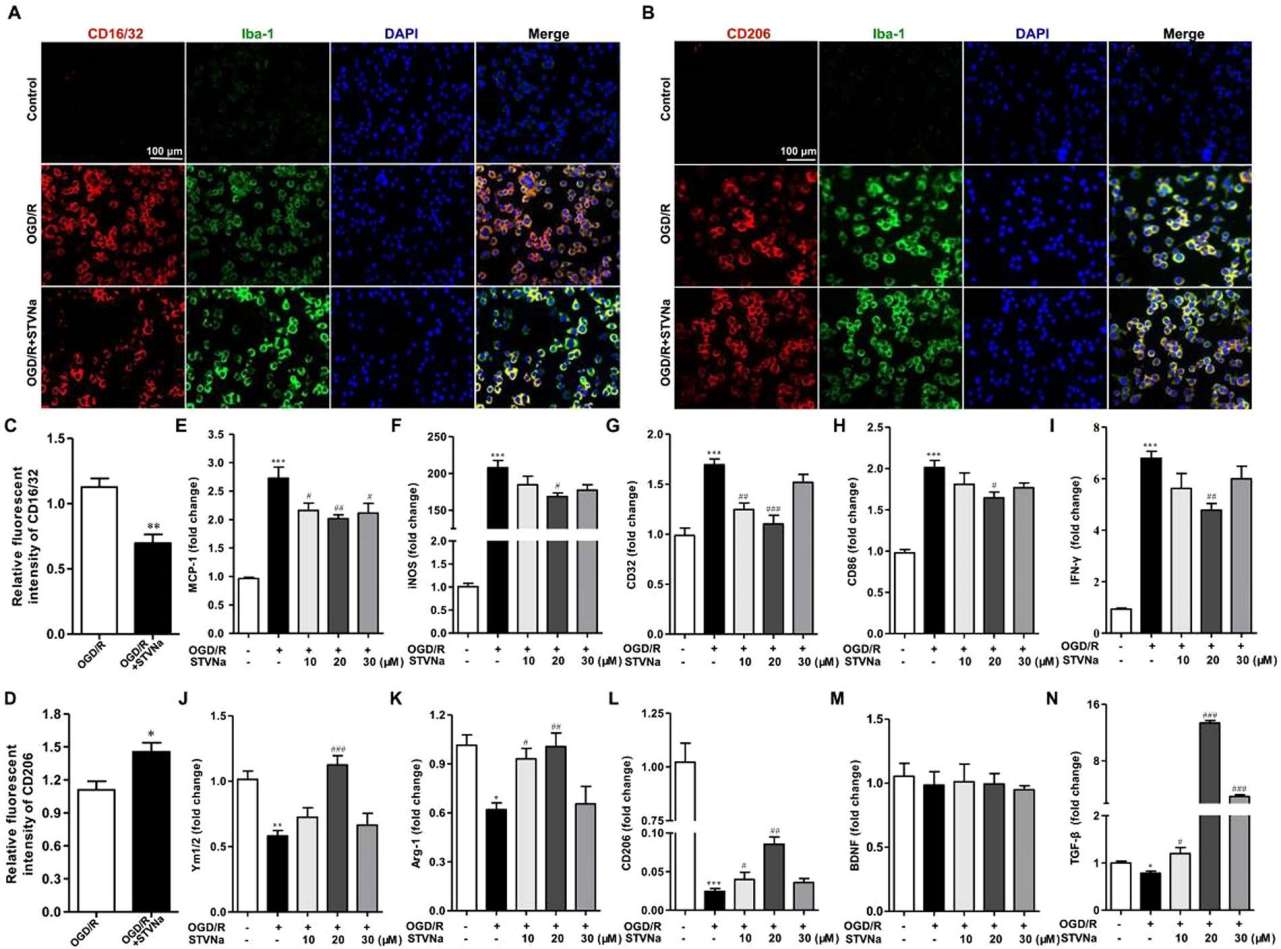
STV-Na inhibits M1 polarization and promotes M2 polarization in BV2 microglial cells after OGD/R. Representative double-immunofluorescence staining for CD16/32 or CD206 and Iba-1 markers in BV2 microglial cells obtained from STV-Na or vehicle treatment after OGD/R, or from Normal. Scale bar: 100 μm. (**A**) BV2 microglial cells co-stained for CD16/32 (M1 marker) (red) and Iba-1(green). (**B**) BV2 microglial cells co-stained for CD206 (M2 marker) (red) and Iba-1 (green). (**C**) Quantification of the percentage of CD16/32^+^/Iba-1^+^ cells among total Iba-1^+^ cells. (**D**) Quantification of the percentage of CD206^+^/Iba-1^+^ cell among total Iba-1^+^ cells. (**E**,**F**,**G**,**H**,**I**,**J**,**K**,**L**,**M**,**N**) RT-PCR analysis of M1 (MCP-1, iNOS, CD32, CD86, IFN-γ) and M2 (Ym1/2, Arg-1, CD206, BDNF, TGF-β) markers in BV2 microglial cells after OGD/R. Data are represented as mean ± SD, ^*^P < 0.05, ^**^P < 0.01, ^***^P < 0.01, versus control; ^#^*P* < 0.05, ^##^*P* < 0.01, versus OGD/R group.

A BV2 microglial cell line was used to further determine the effects of STV-Na on microglial polarization. BV2 microglial cells were treated with OGD/R (4 h OGD + 24 h reperfusion). As shown in Fig. 3E–I, M1 marker (*MCP-1*, *iNOS*, *CD32*, *CD86* and *IFN-γ*) mRNA expression was increased significantly in BV2 microglia cells after OGD/R, but was markedly reduced after treatment with 10, 20, or 30 μM STV-Na. In contrast with this upregulation of M1 markers, M2 markers (*Ym1/2*, *Arg-1*, *CD206*, *BDNF and TGF-β*) mRNA expression were reduced after OGD/R, treatment with 10, 20, or 30 μM STV-Na partially reversed this reduction of CD206 expression (Fig. 3J–N). Immunofluorescence staining further confirmed these results (Fig. 3A–D).

### GAS5 up-regulation is involved in miR-146a-5p down-regulation in ischemic stroke both *in vivo* and *in vitro*

**Figure 4:**
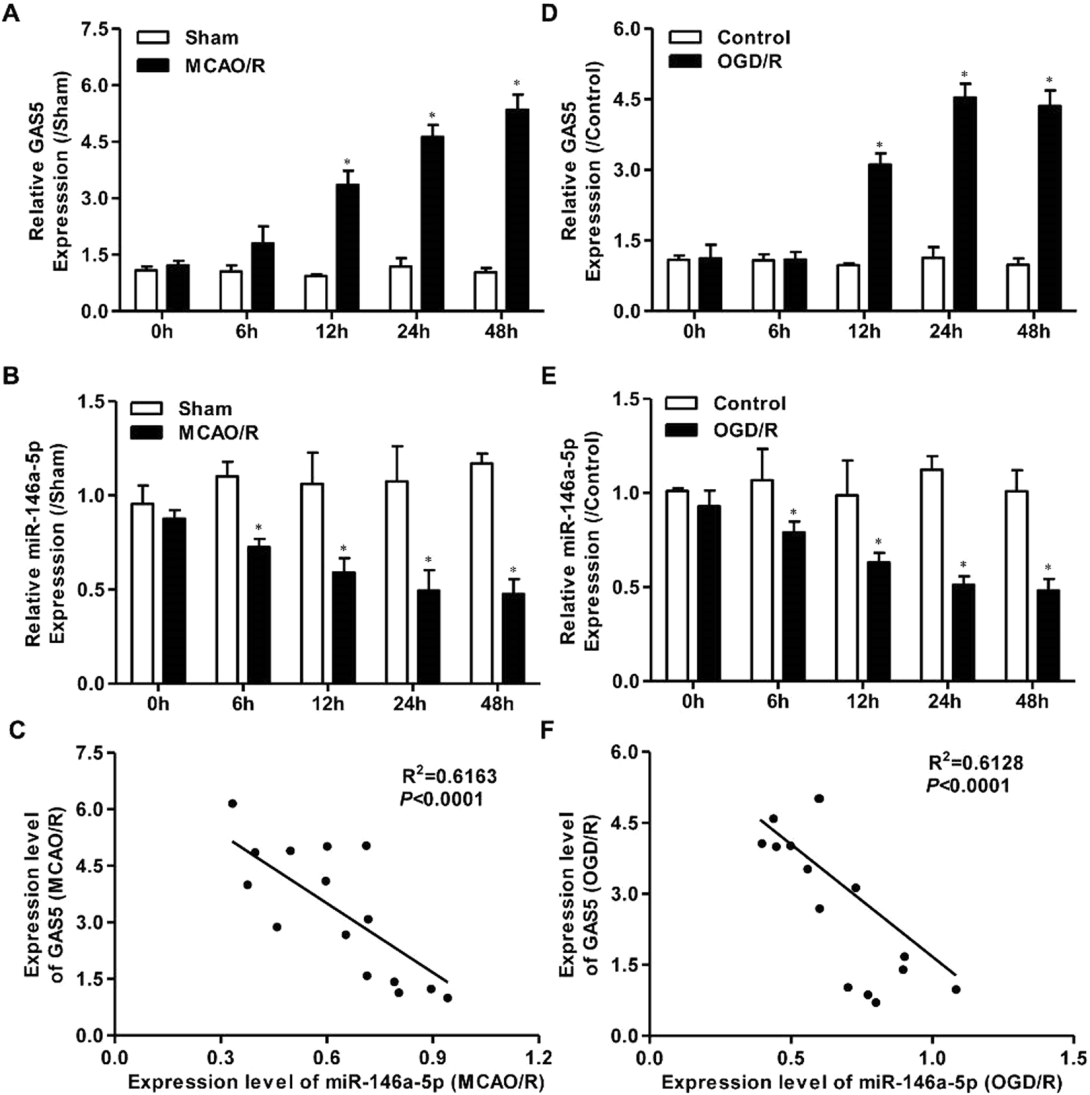
GAS5 up-regulation is involved in miR-146a-5p down-regulation both *in vivo* and *in vitro* models of ischemic stroke. RT-PCR assay for the expression of GAS5 (**A**) and miR-146a-5p (**B**) at each time point in mice subjected to 1 h MCAO and at 0 h, 6 h, 12 h, 24 h, and 48 h of reperfusion (n = 3 per group). RT-PCR was used to detect the expression of GAS5 (**D**) and miR-146a-5p (**E**) in cultured BV2 microglia cells at each time point in BV2 microglial cells subjected to 4 h OGD and at 0 h, 6 h, 12 h, 24 h, and 48 h of reperfusion (n = 3 per group). (**C**) Pearson’s correlation analysis of the relationship between GAS5 and miR-146a-5p in the brain of mice subjected to MCAO/R surgery. (**F**) Pearson’s correlation analysis of the relationship between GAS5 and miR-146a-5p in BV2 microglial cells subjected to OGD/R treatment. Data are represented as mean ± SD, ^*^P < 0.05.

Mice were subjected to MCAO for 1 h followed by reperfusion for different periods of time (0, 6, 12, 24, 48 h). As shown in Fig. 4A, GAS5 expression was markedly enhanced in the MCAO/R group as compared to the sham group. GAS5 levels were also elevated in BV2 microglia subjected to OGD/R as compared to the control group (Fig. 4D). We further found that miR-146a-5p expression was significantly decreased below control levels both in mice subjected to MCAO/R and in BV2 microglia cells exposed to OGD/R (Fig. 4B,E). Finally, we found that GAS5 expression was negatively correlated with miR-146a-5p expression in mice brain tissues after MCAO/R and in BV2 microglia cells exposed to OGD/R (Fig. 4C,F).

### GAS5 inhibition impaired OGD-Induced microglial activation and promoted M1 to M2 phenotype polarization

**Figure 5:**
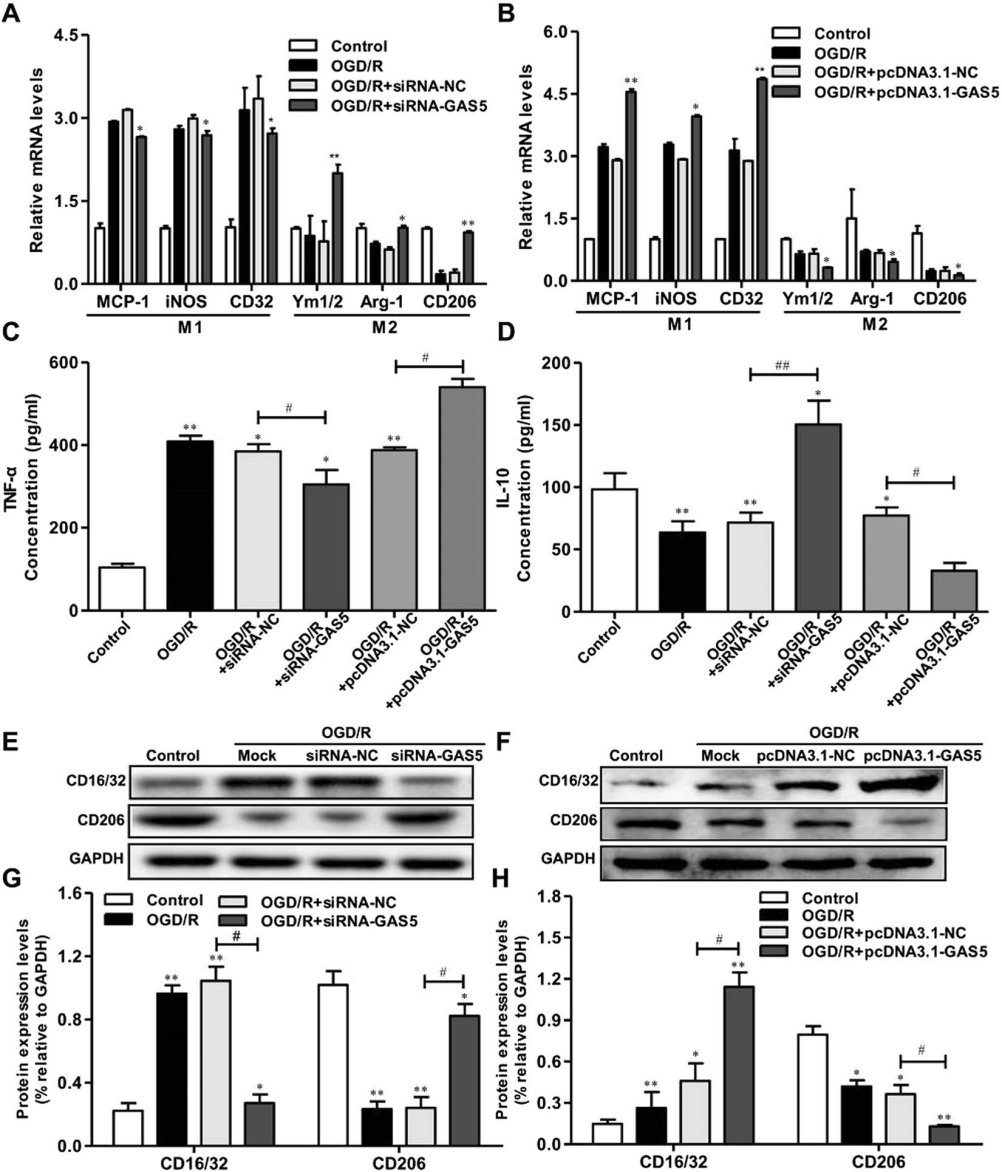
GAS5 suppresses microglial M2 polarization and promotes M1 polarization after OGD/R. RT-PCR analysis of M1 (MCP-1, iNOS, CD32) and M2 (Ym1/2, Arg-1, CD206) markers in BV2 microglial cells transduced with the si-GAS5/siRNA-NC (**A**) or pcDNA3.1-GAS5/pcDNA3.1-NC (**B**). (**C**,**D**) ELISA analysis of TNF-α, and IL-10 in culture supernatants of BV2 microglial cells transduced with the si-GAS5/siRNA-NC or pcDNA3.1-GAS5/pcDNA3.1-NC. (**E**,**G**) Western blotting analysis of CD16/32 and CD206 in microglia transduced with the si-GAS5/siRNA-NC. (**F**,**H**) Western blotting analysis of CD16/32 and CD206 in microglia transduced with the pcDNA3.1-GAS5/pcDNA3.1-NC. Data are represented as mean ± SD, ^*^P < 0.05, ^**^P < 0.01 versus Control; ^#^*P* < 0.05, ^##^*P* < 0.01.

As OGD/R induced M1 polarization of macrophages, to investigate whether GAS5 also directly regulated microglial polarization, we transfected BV2 microglia to overexpress GAS5 (pcDNA3.1-GAS5) or *GAS5* interference (si-GAS5). RT-PCR analyses revealed that GAS5 overexpression in BV2 microglia cells decreased the expression of M2 markers (*Ym-1*, *Arg-1*, and *CD206*), and increased the expression of M1 markers (*MCP-1*, *iNOS*, and *CD32*) (Fig. 5A). Conversely, reduced GAS5 in microglia resulted in increased M2 markers expression and decreased M1 markers expression (Fig. 5B). ELISA and western blotting revealed similar changes in M1 marker (Tumor Necrosis Factor α, TNF-α) and M2 marker (Interleukin 10, IL-10) in the supernatants or protein lysates of microglia overexpressing GAS5 or GAS5 interference microglia (Fig. 5C–H). These results indicate that GAS5 suppressed microglial M2 polarization and promoted M1 polarization following OGD/R.

### GAS5 is a target of MiR-146a-5p and negatively regulates its expression

**Figure 6:**
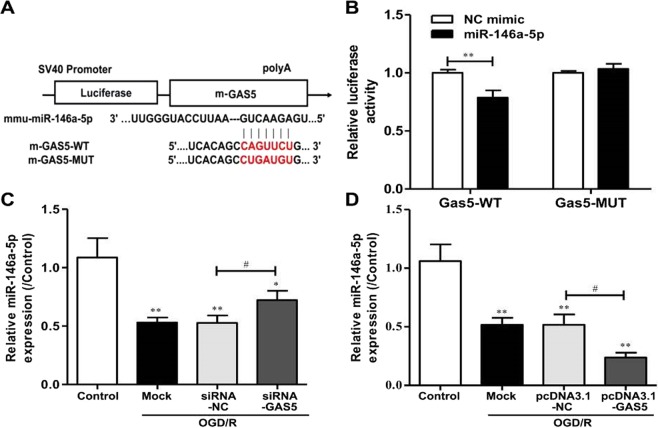
GAS5 is a target of miR-146a-5p and negatively regulates its expression. (**A**) The predicted position of miR-146a-5p binding site on the GAS5 transcript. (**B**) The wide type (GAS5-WT) and mutant GAS5 (GAS5-MUT) were co-transfected with miR-146a-5p mimic or control mimic (NC mimic) into BV2 microglia cells and luciferase activity was detected. (**C**) RT-PCR for the expression of miR-146a-5p in microglia after transfection with si-GAS5/siRNA-NC in the presence of OGD/R exposure. (**D**) RT-PCR for the expression of miR-146a-5p in BV2 microglia cells after transfection with pcDNA3.1-GAS5/pcDNA3.1-NC in the presence of OGD/R exposure. ^*^P < 0.05, ^**^P < 0.01 versus Control; ^#^*P* < 0.05, ^##^*P* < 0.01.

Bioinformatics analyses were conducted using Starbase (http://starbase.sysu.edu.cn), which revealed that *GAS5* contains one conserved target site of miR-146a-5p (Fig. 6A). The dual-luciferase reporter assay revealed that a miR-146a-5p mimic, but not a Negative Control (NC)-mimic, inhibited the luciferase activity of GAS5-WT; however, a miR-146a-5p mimic did not alter the luciferase activity of GAS5-MUT (Fig. 6B). Additionally knockdown or overexpression of *GAS5* in BV2 microglia cells following OGD/R notably increased or decreased miR-146a-5p expression (Fig. 6C,D). These data indicate that GAS5 may have served as a molecular sponge for miR-146a-5p and thus negatively regulated its action.

### MiR-146a-5p directly targeted the 3′UTR of notch1

**Figure 7:**
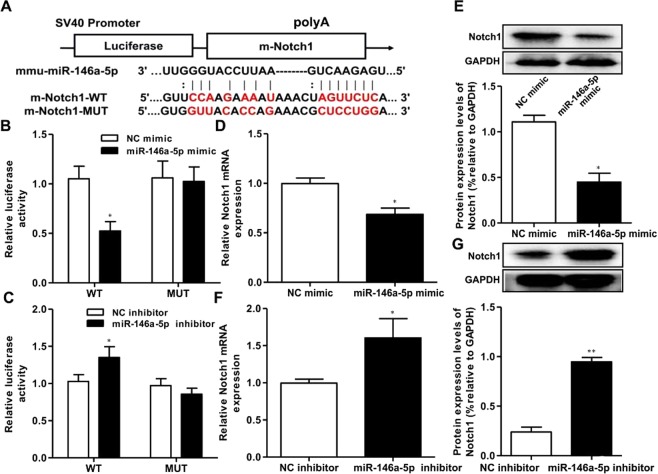
MiR-146a-5p regulate Notch1 expression in BV2 microglia cells. (**A**) The Notch1 was predicted as a target of miR-146a-5p by online bioinformatics methods (Target Scan and microrna.org). (**B**,**D**,**E**) The luciferase activity, the mRNA and protein expression of Notch1 were declined in BV2 microglia cells co-transfected with Notch1-3′UTR-WT and miR-146a-5p mimic. (**C**,**F**,**G**) The luciferase activity and the gene and protein expression of Notch1 were elevated in BV2 microglia cells co-transfected with Notch1-3′UTR-WT and miR-146a-5p inhibitor. Data are represented as mean ± SD, ^*^P < 0.05, ^**^P < 0.01.

Using online bioinformatics analyses (Starbase), *Notch1* was predicted to be a potential target of miR-146a-5p (Fig. 7A). The luciferase reporter assay was used to identify whether the 3′UTR of Notch1 mRNA was a binding target of miR-146a-5p. These results demonstrate that luciferase activity, driven by a miR-146a-5p mimic, was significantly decreased as compared to controls in the Notch1-3′URT-WT group, but that there was no significant change in the Notch1-3′URT-MUT group. RT-PCR and western blot assays revealed that miR-146a-5p upregulation led to a dramatic decrease in Notch1 mRNA and protein expression as compared to controls (Fig. 7B,D,E). In addition, when a miR-146a-5p inhibitor was used to downregulate miR-146a-5p levels, we found the opposite result (Fig. 7C,F,G). These results suggested that the expression of Notch1 mRNA and protein was regulated by miR-146a-5p in BV2 microglia cells, and further that miR-146a-5p directly targeted the 3′UTR of Notch1 in BV2 microglia cells.

### GAS5 regulated Notch1 expression by miR-146a-5p in OGD/R model

**Figure 8:**
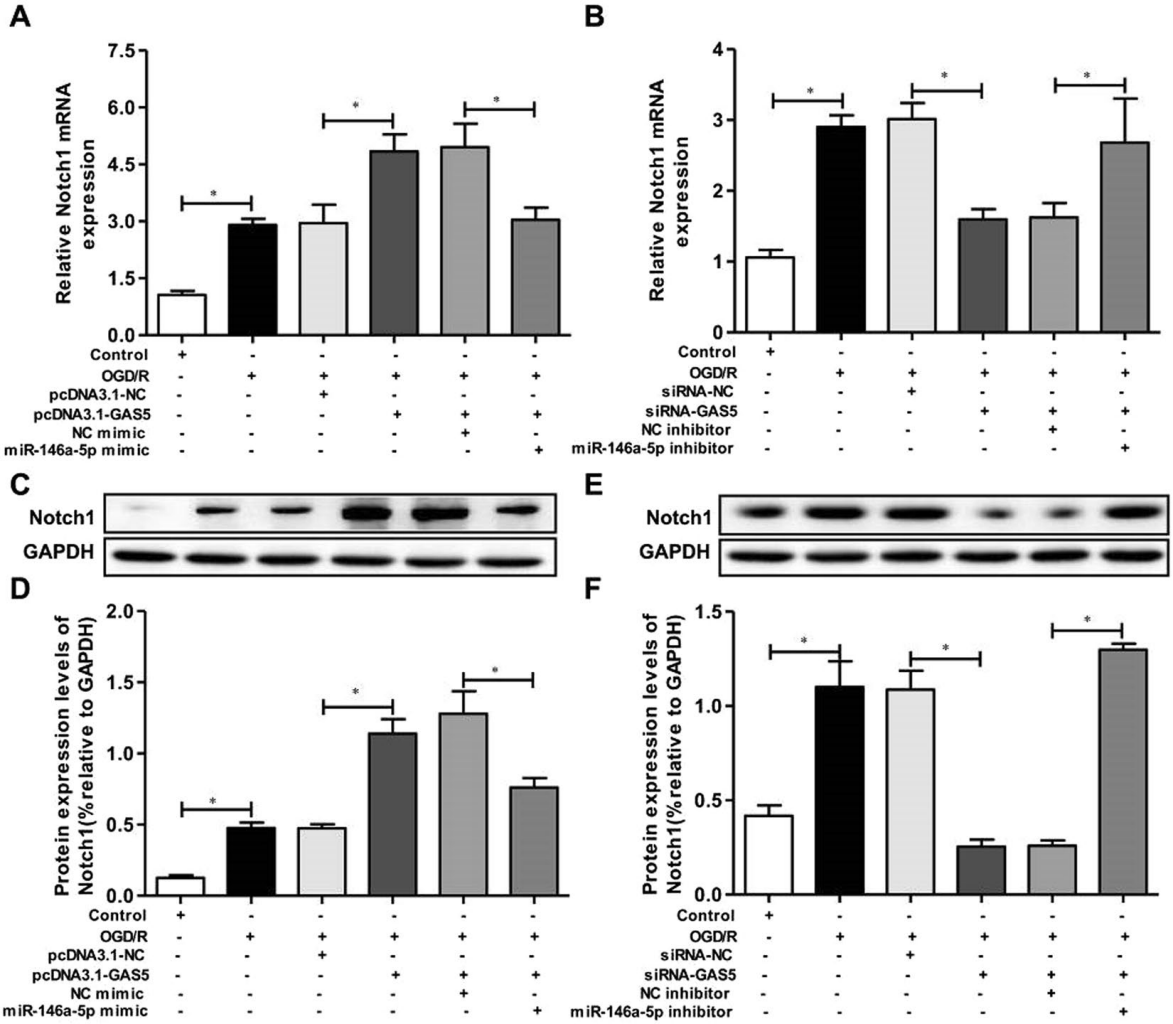
GAS5 regulate Notch1 expression by miR-146a-5p in OGD/R-induced BV2 microglia cells. (**A**,**C**,**D**) The mRNA and protein expression of Notch1 were decreased in OGD/R-induced BV2 microglia cells, and cell transfection of pcDNA3.1-GAS5 upregulated Notch1 expression in OGD/R-induced BV2 microglia cell line. However, miR-146a-5p mimic co-transfected into BV2 microglia cells reversed the effect of pcDNA3.1-GAS5 on Notch1 expression. (**B**,**E**,**F**) The mRNA and protein expression of Notch1 were decreased in BV2 microglia cells transfection of si-GAS5, but miR-146a-5p inhibitor co-transfected BV2 microglia cells reversed the Notch1 expression. Data are represented as mean ± SD, ^*^P < 0.05.

Due to the fact that GAS5 acted as a miR-146a-5p decoy and miR-146a-5p directly targeted the 3′UTR of Notch1, we suspected that GAS5 might regulate Notch1 expression by miR-146a-5p in the OGD/R model. Figure 8A revealed that, Notch1 mRNA levels were significantly reduced in OGD/R-induced BV2 microglia cells, and that OGD/R-induced BV2 cells transfected with pcDNA3.1-GAS5 had increased Notch1 expression. Despite these data, when a miR-146a-5p mimic was co-transfected with pcDNA3.1-GAS5 into BV2 microglia cells, the effect of pcDNA3.1-GAS5 on Notch1 expression was reversed. Next, we performed a GAS5 knockdown using si-GAS5 to verify whether it could downregulate Notch1 expression by miR-146a-5p. As shown in Fig. 8B, Notch1 expression was obviously decreased in cells transfected with si-GAS5, while miR-146a-5p inhibitor co-transfected with si-GAS5 reversed this decreased Notch1 expression. In addition, Notch1 protein expression changes followed those in Notch1 mRNA (Fig. 8C–F). These findings revealed that GAS5 regulated Notch1 expression by miR-146a-5p in the OGD/R model.

### GAS5 altered OGD/R-induced microglial activation by increasing Notch1 expression Via MiR-146a-5p

**Figure 9:**
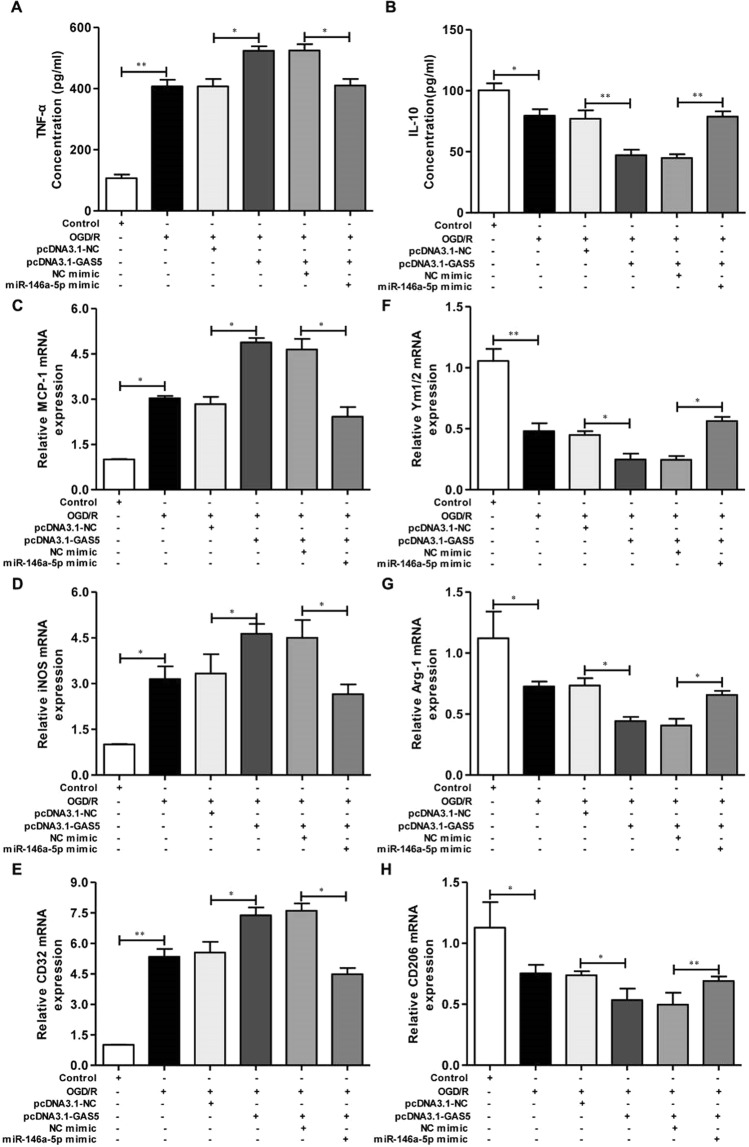
GAS5 affect OGD/R-induced macrophage polarization by miR-146a-5p in BV2 microglia cells. To investigate whether GAS5 could affect OGD/R-induced macrophage polarization by regulating miR-146a-5p. The BV2 microglia cells were transfected or co-transfected with pcDNA3.1-GAS5/pcDNA3.1-NC and miR-146a-5p mimic/NC mimic. (**A**,**B**) The concentrations of TNF-α and IL-10 measured by ELISA. (**C**–**E**) RT-PCR detected the MCP-1, iNOS and CD32 mRNA expression levels. (**F**–**H**) RT-PCR detected the Ym1/2, Arg-1 and CD206 mRNA expression levels. Data are represented as mean ± SD, ^*^P < 0.05, ^**^P < 0.01.

To investigate whether GAS5 impacted OGD/R-induced macrophage polarization by regulating miR-146a-5p, the concentrations of TNF-α and IL-10 and the mRNA expression of M1 (MCP-1, iNOS and CD32) and M2 (Ym1/2, Arg-1 and CD206) markers in BV2 microglia cells transfected or co-transfected with pcDNA3.1-GAS5 and miR-146a-5p mimics were assessed by ELISA and RT-PCR, respectively. As displayed in Fig. 9A, TNF-α concentrations in the OGD/R group were significantly higher than control group levels. However, TNF-α concentrations increased dramatically in cells transfected with pcDNA3.1-GAS5, indicating that pcDNA3.1-GAS5 aggravated the effects of OGD/R on TNF-α production. Despite these data, TNF-α concentrations were markedly decreased when co-transfected with pcDNA3.1-GAS5 and miR-146a-5p mimic, suggesting that miR-146a-5p might reverse the effects of pcDNA3.1-GAS5 on TNF-α concentrations. IL-10 concentrations were opposite those of TNF-α increasing in the OGD/R group, and decreasing dramatically when transfected with pcDNA3.1-GAS5. IL-10 levels also increased in BV2 microglia cells co-transfected with pcDNA3.1-GAS5 and miR-146a-5p mimic, indicating that miR-146a-5p mimic might also reverse the effect of pcDNA3.1-GAS5 on IL-10 production. In addition, we found expected mRNA expression changes in microglia/macrophage polarization genes (Fig. 9C–H). Changes in M1 (*MCP-1*, *iNOS*, and *CD32*) and M2 (*Ym1/2*, *Arg-1*, and *CD206*) markers expression were in keeping with TNF-α and IL-10 concentrations, respectively, suggesting that a miR-146a-5p mimic reversed the effects of pcDNA3.1-GAS5 on gene expression. These data implied that GAS5 reversed the effects of OGD/R on macrophage polarization by miR-146a-5p.

### STV-Na disrupted GAS5/miR-146a-5p to inhibit Notch1 expression after stroke *in vivo* and *in vitro*

**Figure 10:**
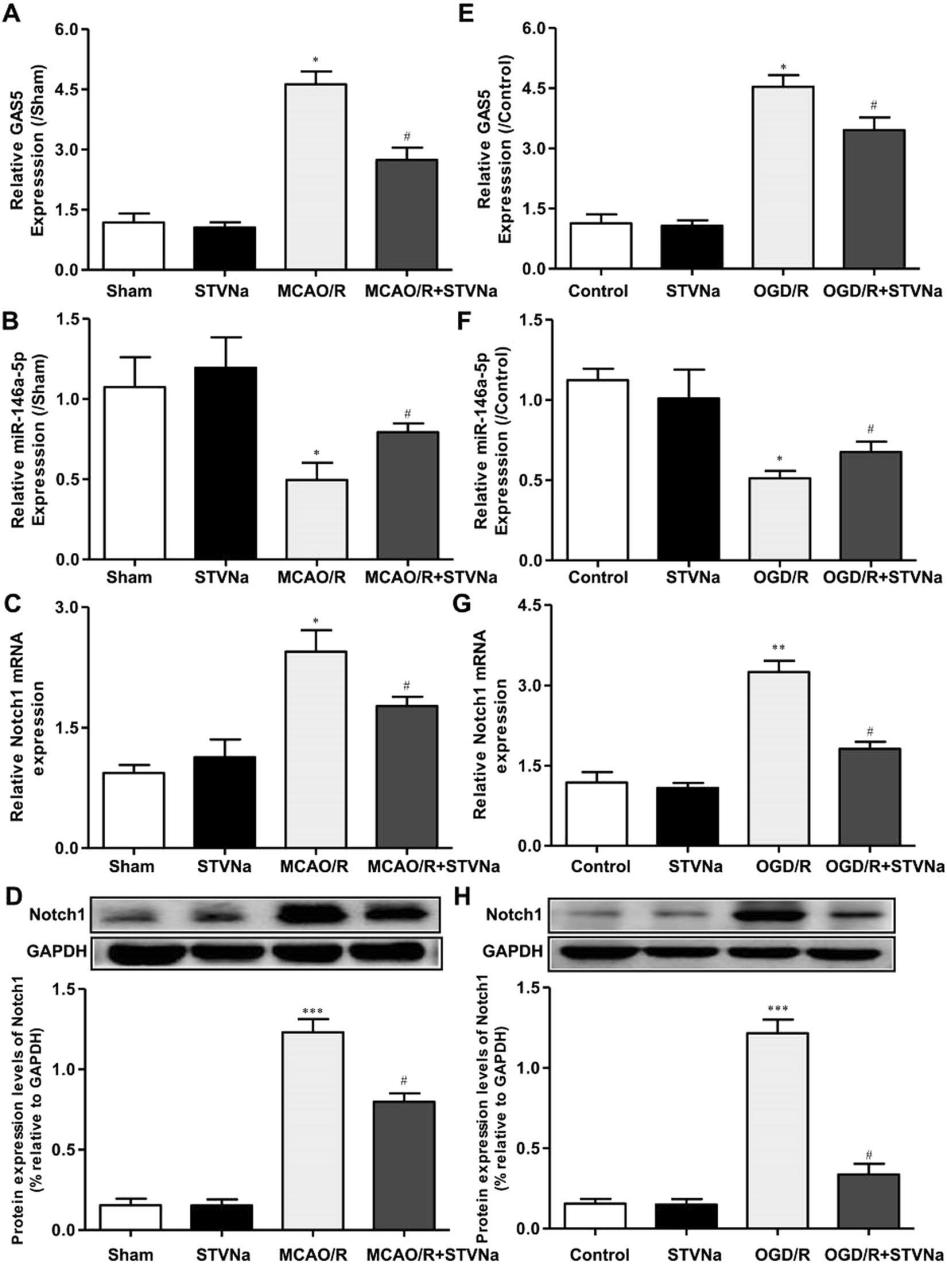
STV-Na disrupted the GAS5/miR-146a-5p to inhibit Notch1 expression after stroke *in vivo* and *in vitro*. RT-PCR assay for the expression of GAS5 (**A**), miR-146a-5p (**B**) and Notch1 (**C**) in mice subjected to MCAO/R (n = 3 per group). RT-PCR was used to detect the expression of GAS5 (**E**), miR-146a-5p (**F**) and Notch1 (**G**) in BV2 microglial cells subjected to OGD/R (n = 3 per group). (**D**,**H**) Western blotting analysis of Notch1 in the ischemic cortex after MCAO/R. (**H**,**J**) Western blotting analysis of Notch1 in BV2 microglia cells after OGD/R. Data are represented as mean ± SD, ^*^P < 0.05, versus Sham or Control; ^#^*P* < 0.05, ^##^*P* < 0.01, versus MCAO/R or OGD/R group.

**Figure 11:**
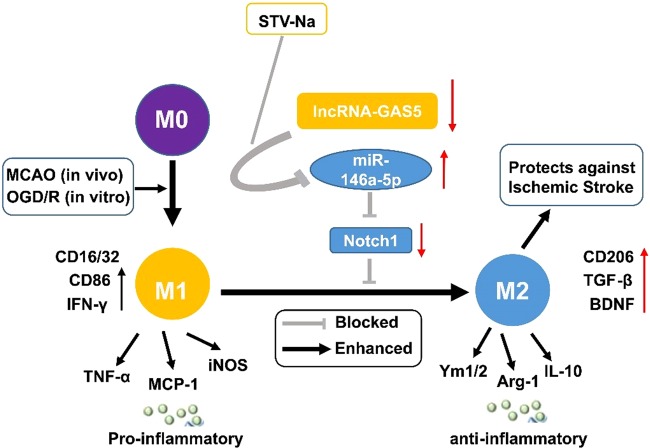
Schematic diagram of STV-Na effect on brain damage post stroke. STV-Na exerts neuroprotection by phenotypic modulation of the microglia/macrophage shift. STV-Na disrupted the GAS5/miR-146a-5p to inhibit Notch1 expression after stroke *in vivo* and *in vitro*. Thus, expression of pro-inflammatory cytokines was suppressed and the polarization of microglia/macrophages was shifted to the M2 phenotype.

STV-Na had strong effects on cerebral ischemia. We also measured the gene expression of GAS5 and the protein expression of Notch1 *in vivo* and *in vitro*, and found that both decreased with 30 mg/kg of STV-Na (Fig. 10). Furthermore, mRNA levels of miR-146a-5p were higher *in vivo* and *in vitro* treated with STV-Na compared to the model group. These results suggest that STV-Na protects against ischemic stroke injury by titrating microglia/macrophage polarization via GAS5/miR-146-5p sponge (Fig. 11).

## Discussion

STV-Na is a beyerane diterpene obtained from the acid hydrolysis of stevioside, which has been used in traditional medicine for hundreds of years^35^. In the present study, we demonstrated that STV-Na treatment not only decreased infarct volumes, but also improved multiple components of neurological functioning at acute and chronic time points following ischemic stroke. Moreover, we discovered that STV-Na shifted microglia/macrophage polarization toward neuroprotective and tissue-reparative M2 phenotypes in the ischemic brain. *In vitro* studies in a microglial cell line further confirmed this direct effect of STV-Na on microglial polarization (Supplementary material). Finally, GAS5/miR-146a-5p sponges were further disrupted by STV-Na, decreasing brain damage.

An important component of the central nervous system (CNS), microglia are the major immune cells of the brain, serving as the first line of defense against brain injury^36^. Peripheral macrophages also infiltrate the CNS between hours and days following tMCAO, serving as a bridge between the CNS and peripheral immune system. Activated microglia/macrophages may further serve both protective and deleterious roles, depending on their phenotypes^37^. For instance, the M1 phenotype is characterized by the production and release of pro-inflammatory mediators (*TNF-α*, *MCP-1*, and *iNOS*), which accelerate cell death and aggravate inflammation. In contrast, the M2 phenotype improves neuronal survival and tissue repair by producing anti-inflammatory mediators (*CD206*, *Ym1/2*, and *Arg1*)^18,38^. This lack of necessary endogenous signals for M2 induction is known to worsen outcomes following cerebral ischemia. It was further confirmed *in vitro* that M1 microglia exacerbate neuronal damage, whereas M2 counterparts prevent neuronal death after ischemia^39^. Thus, shifting microglia from the M1 to the M2 phenotype may be an effective therapeutic strategy for decreasing ischemic stroke damage in the brain. Notably, we discovered here that STV-Na shifts microglia/macrophage polarization towards the neuroprotective and tissue-reparative M2 phenotype in the ischemic brain. Studies in a microglial cell line further confirmed a direct effect of STV-Na on microglial polarization *in vitro* and decreased cerebral inflammation in the ischemic brain *in vivo*.

Once considered to be transcriptional “noise”, the functional roles of lncRNAs in neurological disorders are today widely recognized^40^. GAS5 is a functional lncRNA that was identified decades ago and is abnormally expressed in many types of tumors and inhibits T-cell proliferation^41,42^. Furthermore, increasing number of studies have found that *GAS5* plays critical role in cerebral ischemia. For instance, hypoxia/ischemia significantly up-regulates *GAS5* expression and GAS5 knockdown protects hippocampal neurons from hypoxia/ischemia-induced brain injury, likely acting as a neuroprotective agent, at least partially via the modulation of antioxidant enzyme activity^43^. GAS5 is also closely linked to the polarization of microglia in central nervous system disease^44^. For instance, M2b macrophage polarization is accompanied by reduced long noncoding RNA GAS5^28^. LncRNAs may epigenetically regulate gene expression by competing for shared miRNA response elements, thereby acting as a natural miRNA sponge to reduces endogenous miRNAs binding to target genes^45^. We predicted here, using a bioinformatics approach, that *GAS5* might serve this role as miR-146a-5p shares complementary binding sites at a 3′UTR, which was confirmed by the luciferase assay. The dual-luciferase reporter assay confirmed that GAS5 contains one direct binding site for miR-146a-5p in its 3′ UTR, which was sponged up by GAS5 *in vitro*. Loss-of-function and rescue experiments further revealed that miR-146a-5p regulated *GAS5* gene expression in cells. We also confirmed that STV-Na disrupted the GAS5/miR-146a-5p sponge to promote M2 microglial polarization and inhibit microglia-mediated pro-inflammatory responsivity.

Notch1 acts as an architectural transcription factor by introducing structural alterations in chromatin^46^. Several studies have found a relationship between Notch signaling and macrophage polarization^47,48^. For instance, the activation of Notch1 increase M1 macrophage immune responsivity. In the present study, we found that the expression of Notch1 protein was decreased in BV2 microglia cells when treated with STV-Na. We further demonstrated that the expression of Notch1, a target of miR-146a-5p, was regulated by miR-146a-5p after OGD/R. Given these findings, we put forward a proposed model whereby STV-Na disrupts the GAS5/miR-146a-5p sponge, leading to Notch1 induction and the subsequent inhibition of microglia-mediated pro-inflammatory responses. GAS5 likely interacts with and sequesters miR-146a-5p in the nucleus. This sequestering was inhibited STV-Na, causing miR-146a-5p to bind to Notch1 3′UTR and thereby inhibiting M1 polarization after OGD/R (Fig. 6D). Therefore, we propose that STV-Na disrupted the GAS5/miR-146a-5p sponge, leading to Notch1 induction, which in turn promoted M1 to M2 phenotype polarization.

In summary, using a series of *in vitro* and *in vivo* experiments, we found that STV-Na is neuroprotective against ischemia/reperfusion injury in mice. More specifically, STV-Na promoted M2 microglial polarization via disruption of the GAS5/miR-142-3p sponge (Fig. 11). Our data are consistent with previous studies and further highlight the potential role of STV-Na as a therapeutic agent for ischemic stroke. Moreover, mechanistic investigation of natural compounds such as STV-Na may result in the development of safer and more potent neuroprotective agents. Further studies, including clinical trials, are needed to confirm the safety and efficacy of this compound as an adjuvant to traditional chemotherapeutic regimens.

## Materials and Methods

### Animals

Male C57BL/6 mice (20–25 g, 7 weeks old) were purchased from the Animal Research Centre of Guangzhou University of Chinese Medicine (Guangzhou, China). The mice were housed in a temperature-controlled environment (25 °C ± 2 °C), with a 12-hour light/dark cycle and free access to food and water. All experiments were performed according to animal use guidelines and approved by the Institutional Animal Care and Use Committee of Guangdong Pharmaceutical University.

### Focal cerebral ischemia model

Focal cerebral ischemia was induced in mice by intraluminal middle cerebral artery occlusion (MCAO) as described previously^49^. Briefly, male mice were anesthetized with 1.5%–3% isoflurane. Under an operating microscope, the right common carotid artery (CCA), and external and internal carotid arteries were surgically exposed through a neck incision. A 6-0 silicon-coated nylon filament was introduced into the CCA and advanced into the internal carotid artery until the tip reached the origin of the middle cerebral artery (MCA), which was detected by a mild increase in resistance. Occlusion of the right MCA was induced either transiently for 60 min followed by a reperfusion period of 1, 3, 7, 14 d (MCAO/R). Sham-operated mice received the same experimental surgery without a filament being inserted into the MCA.

### STV-Na administration

STV-Na was obtained from the Chemical Development Laboratories of Key Biological Pharmaceutical Company (Dongguan, China). In the dose-response experiment, mice were assigned to 1 of the following 8 groups: sham (n = 6), vehicle (n = 6), STV-Na (15, 30, 45, and 60 mg/kg, n = 6 per group). Dose volumes were maintained at approximately 0.12 ml. Moreover, in MCAO/R-induced polarization experiments, 30 mg/kg dose of STV-Na was administered at 3, 7 or 14 days after reperfusion (n = 6 per group). Sham and vehicle were delivered 1 hour after MCAO (n = 6 per group). Mice were randomly assigned to each experimental group before MCAO/R.

### Behavioral tests and infarct size assessment

Following cerebral ischemia, mice were also tested for neurological deficits and each mouse completed a set of tests in order to obtain a modified Neurological Severity Score (mNSS), thus comprehensively evaluating neurological function 1, 3, 7, and 14 d after MCAO. The mNSS comprised motor, sensory, reflex, and balance tests; details of the points system can be found in previous studies^50^. Mice were sacrificed by intraperitoneal injection with 400 mg/kg of chloral hydrate after evaluating infarct size, details of the points system can be found in previous studies^30^. The investigator administering the behavior tests and infarct size were not involved in the surgeries and was blinded to the experimental groups.

### BV2 cell culture and OGD treatment

The BV2 microglia cell line were purchased from American Type Culture Collection (ATCC, Manassas, VA, USA). The cells were cultured in Dulbecco modified Eagle medium (DMEM; Gibco, Grand island, NY, USA) supplemented with 10% (v/v) heat-inactivated fetal bovine serum (FBS), 100 μg/mL streptomycin, and 100 U/mL penicillin (Hyclone, Logan, UT, USA). Cells were incubated at 37 °C in a humidified atmosphere containing 5% CO_2_ and the medium was changed every 2 days. Cells were incubated for a further 24 hours in a humidified incubator. Cells in the OGD group were cultured in an ischemia-mimetic solution and kept for 4 hours at 37 °C in a hypoxic incubator chamber filled with 95% N2/5% CO2. Cells were then transferred to normal culture medium for 24 hours and kept at 37 °C in an incubator with 5% CO2 for reperfusion.

### Immunofluorescence staining

*In vivo* experiment, the brain slices (5 μm) were fixed in 4% paraformaldehyde for 10 min, blocked with 5% donkey serum for 1 h, and then incubated with CD16/32 and CD206 primary antibody (Abcam, 1:100) overnight at 4 °C^51^. The slices were washed for 3 times and incubated with the Alexa Fluor 594-conjugated secondary antibody (Invitrogen, 1:200) for 1 h at room temperature. Brain slices were mounted and covers lipped using the fluorescent mounting medium. All slices were observed by a fluorescent microscope. *In vitro* experiment, Immunofluorescence staining was performed according to the protocol previously described^52^.

### RNA preparation and real-time PCR (RT-PCR)

Total RNA was extracted from the cortex of C57BL/6 mice or from the BV2 microglia cell line (ATCC, Manassas, VA, USA) by the RNaEXTM Total RNA Isolation Kit (Generay, Shanghai, China) according to the manufacturer’s instructions. The concentration of RNA was determined by NanoDrop-2000 spectrophotometry (NanoDrop2000, Thermo, USA). Reverse transcription (RT) was performed using the miRNA cDNA Synthesis kit (CWBIO, Beijing, China). The 20 μl RT reaction solutions containing 4 μl 160–320 ng RNA, 4 μl 5X SuperRT buffer, 1 μl enzyme mix, 3 μl RT primer, 1 μl UltraPure dNTP Mix, 0.5 μl SuperRT, and 7.5 μl RNase-Free water were incubated for 50 min at 42 °C followed by heat inactivation of the reverse transcriptase for 5 min at 85 °C.

The RT-PCR amplification was performed with an ABI7500 (American Applied Biosystems Inc, Carlsbad, CA, USA) using the miRNA and mRNA qPCR assay kit (CWBIO, Beijing, China). The detail of the procedures can be found in previous studies^32^. Sequences of primers for RT-PCR were designed for qPCR by Generay (the oligo sequences are shown in Table 1).

**Table 1:** RT-PCR primers used in the study.

Genes		Primers 5′-3′
MCP-1	Forward	ATGCAGGTCCCTGTCATGC
	Reverse	GCTTGAGGTGGTTGTGGAG
iNOS	Forward	CAAGCACCTTGGAAGAGGAG
	Reverse	AAGGCCAAACACAGCATACC
CD32	Forward	AATCCTGCCGTTCCTACTGATC
	Reverse	GTGTCACCGTGTCTTCCTTGAG
Ym1/2	Forward	CAGGGTAATGAGTGGGTTGG
	Reverse	CACGGCACCTCCTAAATTGT
Arg-1	Forward	TCACCTGAGCTTTGATGTCG
	Reverse	CTGAAAGGAGCCCTGTCTTG
CD206	Forward	CAAGGAAGGTTGGCATTTGT
	Reverse	CCTTTCAGTCCTTTGCAAGC
GAPDH	Forward	AGGTCGGTGTGAACGGATTTG
	Reverse	GGGGTCGTTGATGGCAACA

### Western blotting

Samples from the mouse cerebral cortex or BV2 cultures were homogenized in lysis buffers, and total protein was isolated as described previously^53^. Protein was loaded onto an SDS polyacrylamide gel. After electrophoresis, the protein was transferred onto a PVDF membrane (Millipore, Billerica, MA, USA). The membrane was incubated with the following antibodies at 4 °C overnight: CD16/32, CD206, Notch1 (1:500, Sigma, St Louis, MO, USA) and GAPDH (1:2000, Millipore) and then with the HRP-conjugated secondary antibodies for 45 min at room temperature, GAPDH was used as an internal control. Next, the membranes were incubated with and detected using an enhanced chemiluminescence detection system (Pierce, Rockford, USA).

### Cell transfection

For siRNA knockdown of GAS5, BV2 microglia cells were transfected with GAS5 siRNA (si-GAS5) or control siRNA. For miR-146a-5p knockdown, BV2 microglia cells were transfected with miR-146a-5p with negative control (NC inhibitor) as control. For overexpressing GAS5, recombinant pcDNA3.1 plasmid of GAS5 gene was transfected into BV2 microglia cells with pcDNA3.1-NC empty plasmid as control. For miR-146a-5p overexpression, the cells were transfected with miR-146a-5p mimic as compared with negative control (NC mimic). All transfections were carried out using lipofectamine2000 according to manufacturer guidelines and cells were harvested after 48 h for subsequent experiments.

### Luciferase reporter assay

The bioinformatics website (http://starbase.sysu.edu.cn) was adopted to analyze binding sites of GAS5 and miR-146a-5p. The fragment from GAS5 3′-untranslated region (3′UTR) containing the predicted miR-146a-5p binding site was cloned into pmirGLO vector (RiboBio, Guangzhou, China) to form the reporter vector GAS5-wild-type (GAS5-WT). To mutate the putative binding site of miR-146a-5p in GAS5 3′UTR, site-mutations were performed to generate GAS5-mutated-type (GAS5-MUT). Then the vectors (GAS5-WT or GAS5-MUT) and miR-146a-5p mimics or scramble miRNAs were co-transfected into cells, and Dual Luciferase Reporter Assay System (RiboBio, Guangzhou, China) was used for testing luciferase activity. The luciferase reporter assay of miR-146a-5p and Notch1 was the same as the above steps.

### ELISA measurements

Cytokine production (TNF-α, IL-10) in the BV2 culture media was determined by ELISA (Beyotime Co., Ltd., Shanghai, China), according to the manufacturer’s instructions. Each sample was assayed in triplicate.

### Statistical analysis

Data are presented as the mean ± standard deviation (SD). The Student’s t-test or one-way ANOVA followed by Tukey’s multiple comparison tests was used to determine statistical significance between groups with SPSS 20.0 software. Correlations between the expression of GAS5 and miR-146a-5p were performed by Pearson’s correlation. *P* < 0.05 was considered statistically significant.

## Supplementary information

Supplementary material
