# Supplementary material for: Isosteviol Sodium Protects against Ischemic Stroke by Modulating Microglia/Macrophage Polarization via Disruption of GAS5/miR-146a-5p sponge
Source: Sci Rep. 2019 Aug 21;9:12221. doi: 10.1038/s41598-019-48759-0 (PMC6704123; doi:10.1038/s41598-019-48759-0)
Supplement: Supplementary file 1 — Supplementary material [file 41598_2019_48759_MOESM1_ESM.pdf]

Isosteviol Sodium Protects against Ischemic Stroke by Modulating Microglia/Macrophage Polarization via Disruption of GAS5/miR-146a-5p sponge

Hao Zhang, Minyi Lu, Xiaofeng Zhang, Yihe Kuai, Ying Mei, Qiwen Tan, Kailun Zhong, Xiaoou Sun*, and Wen Tan*

Institute of Biomedical and Pharmaceutical Sciences, Guangdong University of Technology, Guangzhou, China

* Corresponding author: Xiaoou Sun, Ph.D. Wen Tan, MD., Ph.D.

Xiaoou Sun, Institute of Biomedical and Pharmaceutical sciences, Guangdong University of Technology, Guangzhou 510006, China. xiaoousun@gdut.edu.cn, Tel: 13539850005

Wen Tan, Institute of Biomedical and Pharmaceutical sciences, Guangdong University of Technology, Guangzhou 510006, China. went@gdut.edu.cn, Tel: 13928954505

Supporting Information 1. Western blotting results.

1) This file contains the original blot images of all the samples.
2) Guarantee 3 independent repeated experiments for every case.
3) The framed areas were used in the main text.

Figure 5E

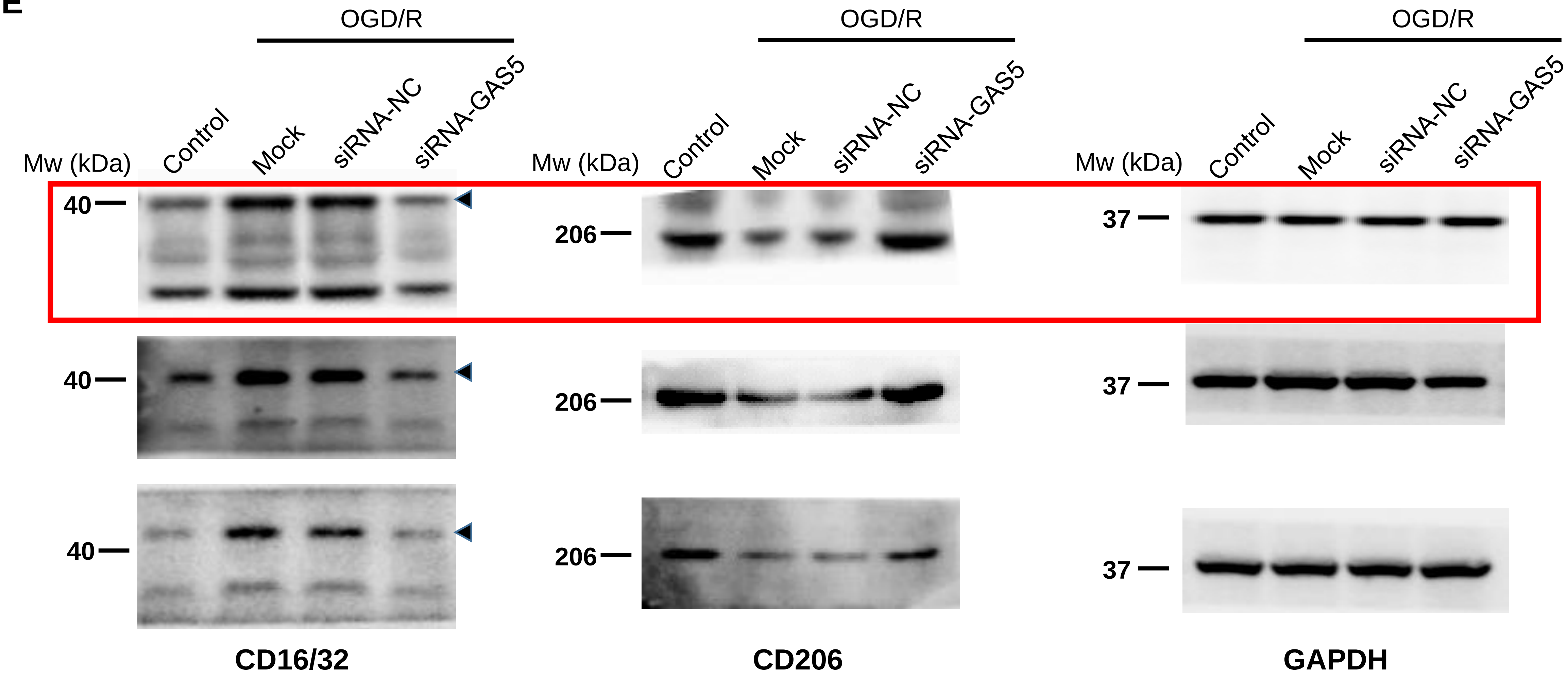

OGD/R
OGD/R
OGD/R
Mw (kDa)
Control
Mock
siRNA-NC
siRNA-GAS5
Mw (kDa)
Control
Mock
siRNA-NC
siRNA-GAS5
Mw (kDa)
Control
Mock
siRNA-NC
siRNA-GAS5
40
206
37
40
206
37
40
206
37
CD16/32
CD206
GAPDH

Figure 5F

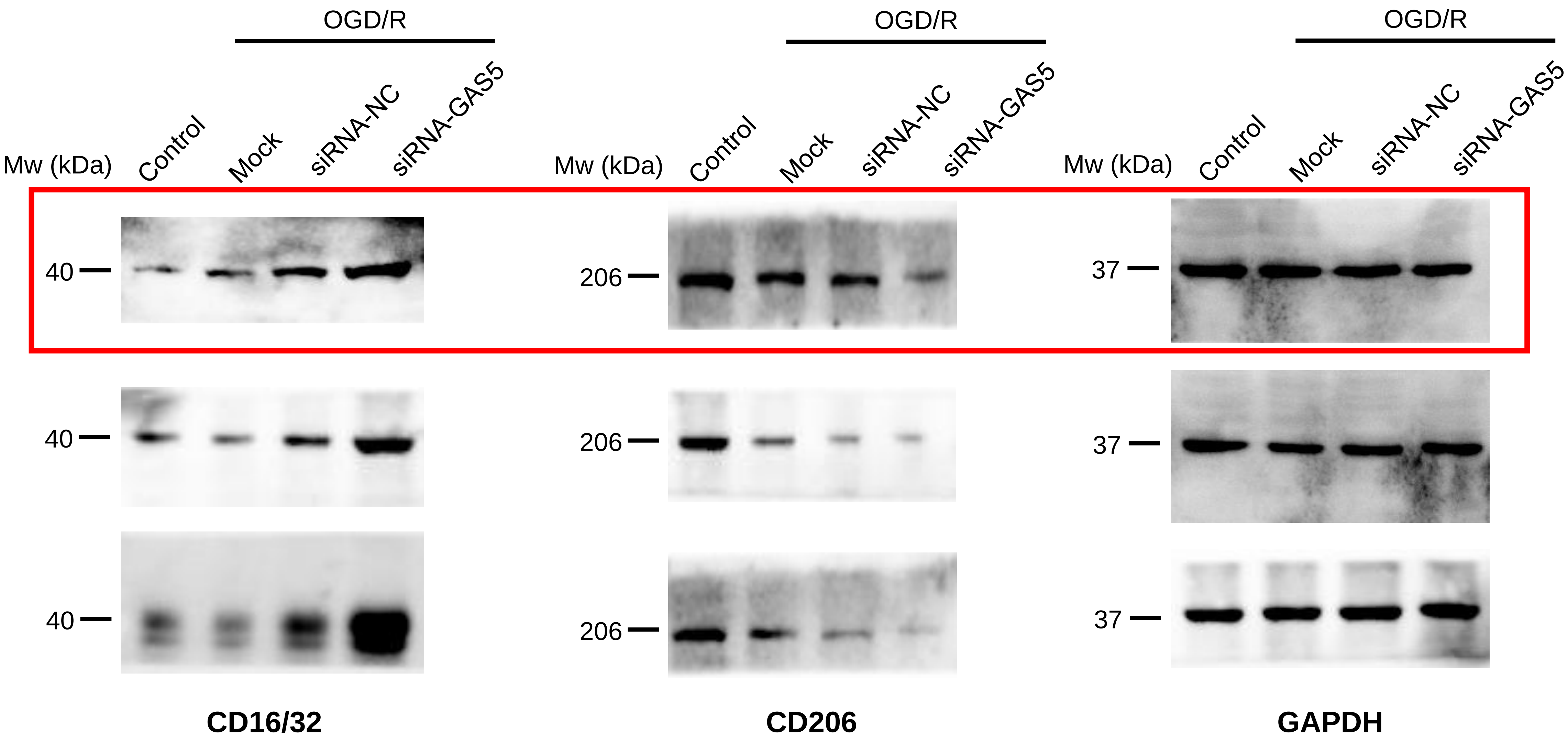

OGD/R
Mw (kDa)
Control
Mock
siRNA-NC
siRNA-GAS5
40
206
37
CD16/32
CD206
GAPDH

Figure 7E

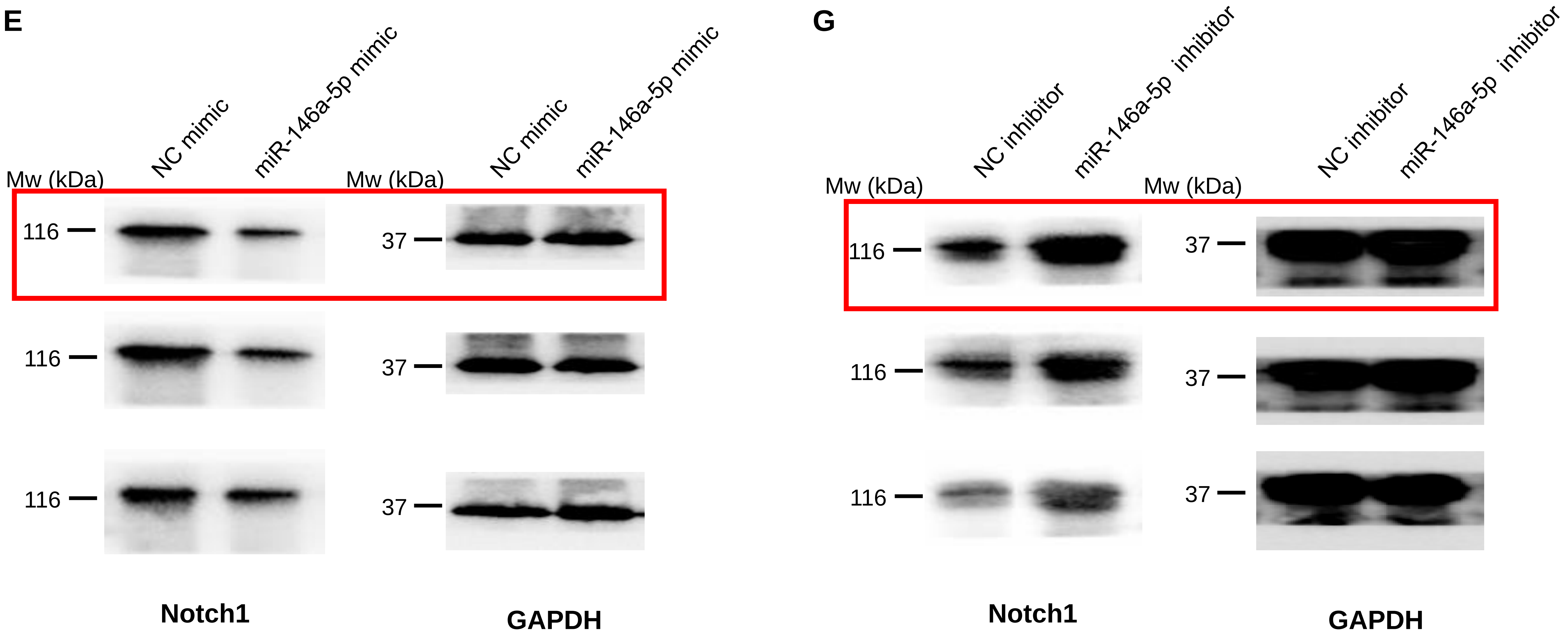
E
NC mimic
miR-146a-5p mimic
NC mimic
miR-146a-5p mimic
Mw (kDa)
Mw (kDa)
116
37
116
37
116
37
Notch1
GAPDH
G
NC inhibitor
miR-146a-5p inhibitor
NC inhibitor
miR-146a-5p inhibitor
Mw (kDa)
Mw (kDa)
116
37
116
37
116
37
Notch1
GAPDH

Figure 8C

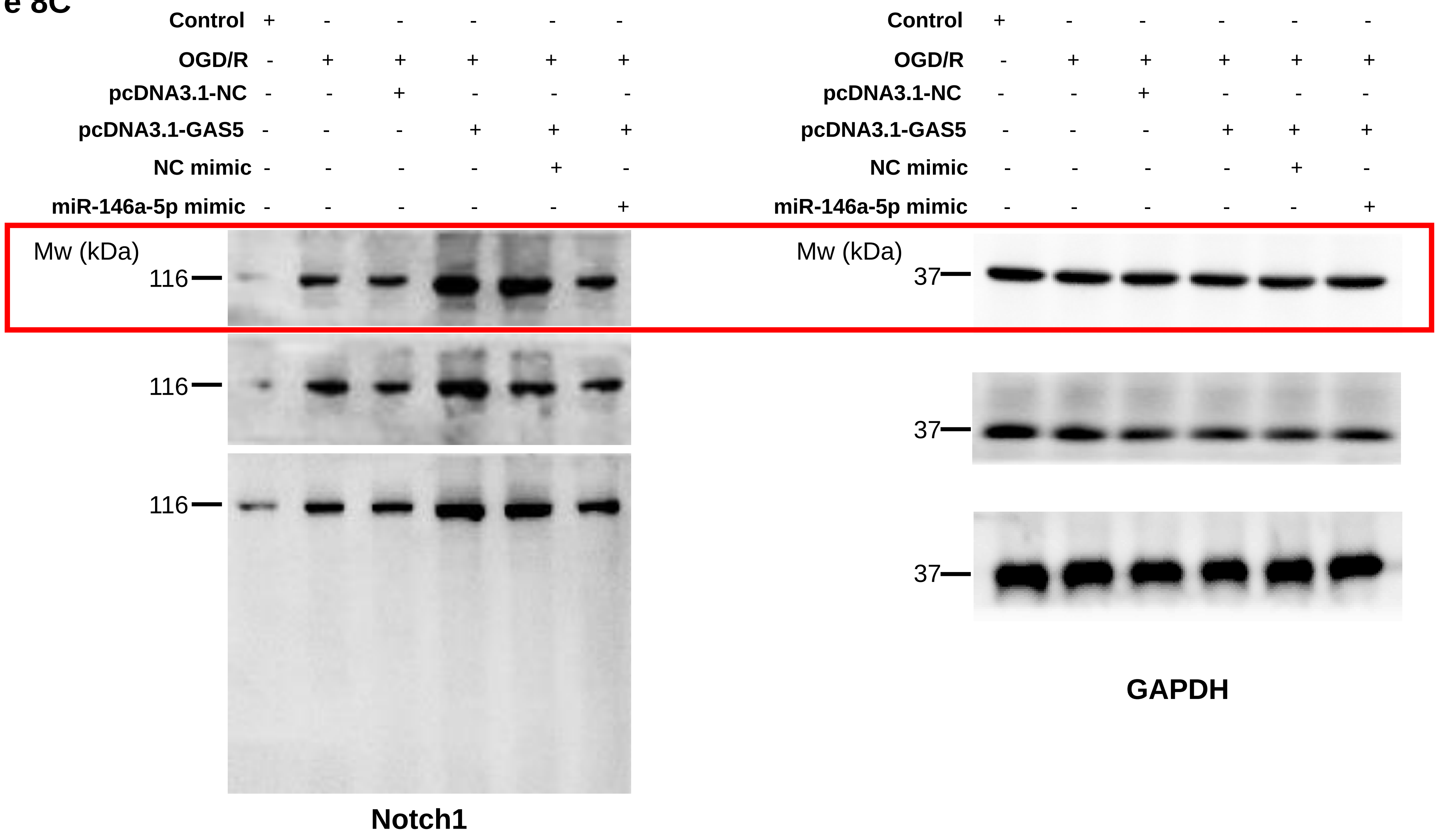

Control + - - - - -
OGD/R - + + + + +
pcDNA3.1-NC - - + - - -
pcDNA3.1-GAS5 - - - + + +
NC mimic - - - - + -
miR-146a-5p mimic - - - - - +
Mw (kDa)
116
116
116
Notch1
Control + - - - - -
OGD/R - + + + + +
pcDNA3.1-NC - - + - - -
pcDNA3.1-GAS5 - - - + + +
NC mimic - - - - + -
miR-146a-5p mimic - - - - - +
Mw (kDa)
37
37
37
GAPDH

Figure 8E

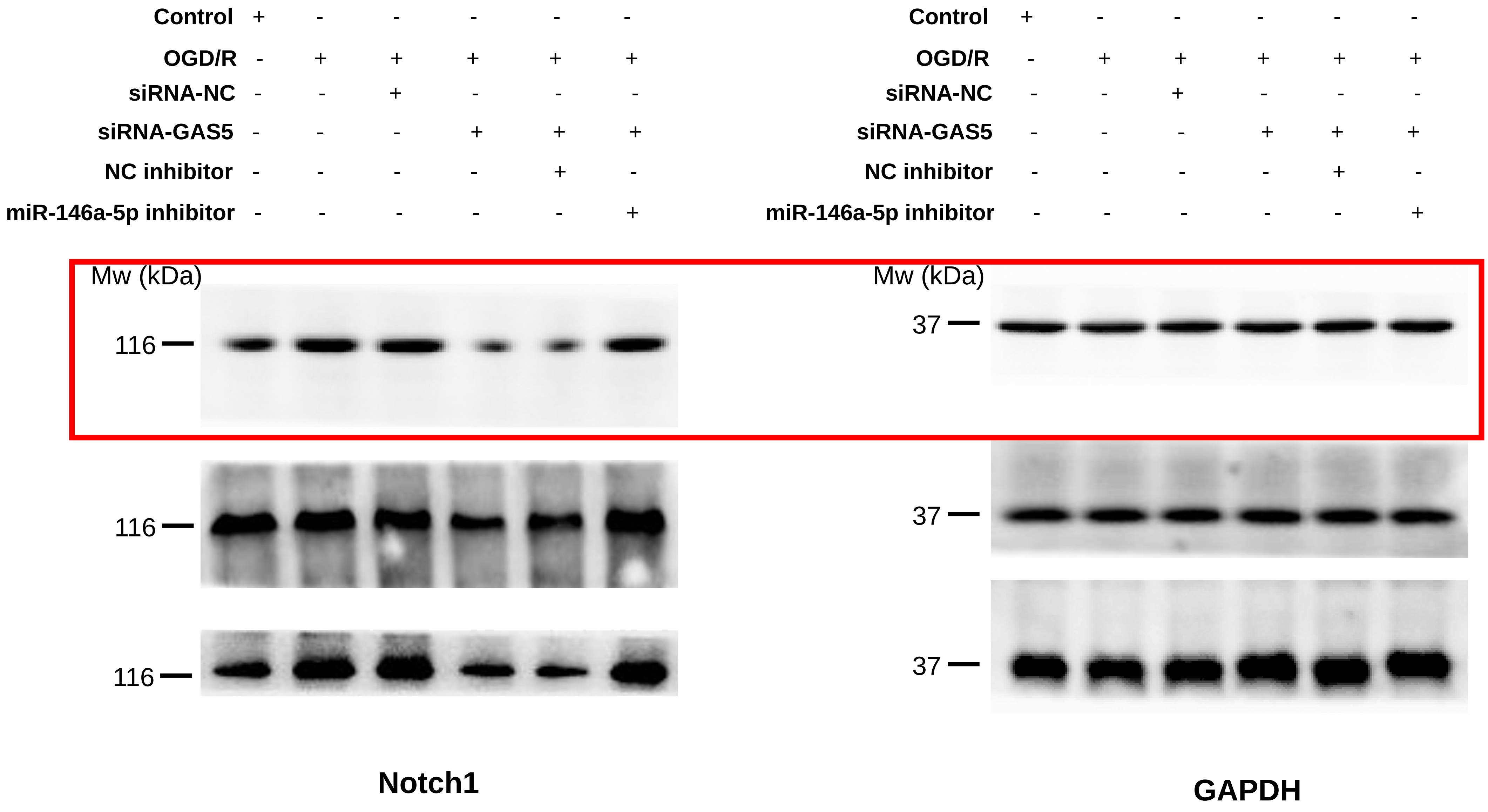
Control + - - - - -
OGD/R - + + + + +
siRNA-NC - - + - - -
siRNA-GAS5 - - - + + +
NC inhibitor - - - - + -
miR-146a-5p inhibitor - - - - - +
Control + - - - - -
OGD/R - + + + + +
siRNA-NC - - + - - -
siRNA-GAS5 - - - + + +
NC inhibitor - - - - + -
miR-146a-5p inhibitor - - - - - +
Mw (kDa)
116
116
116
Mw (kDa)
37
37
37
Notch1
GAPDH

Figure 10

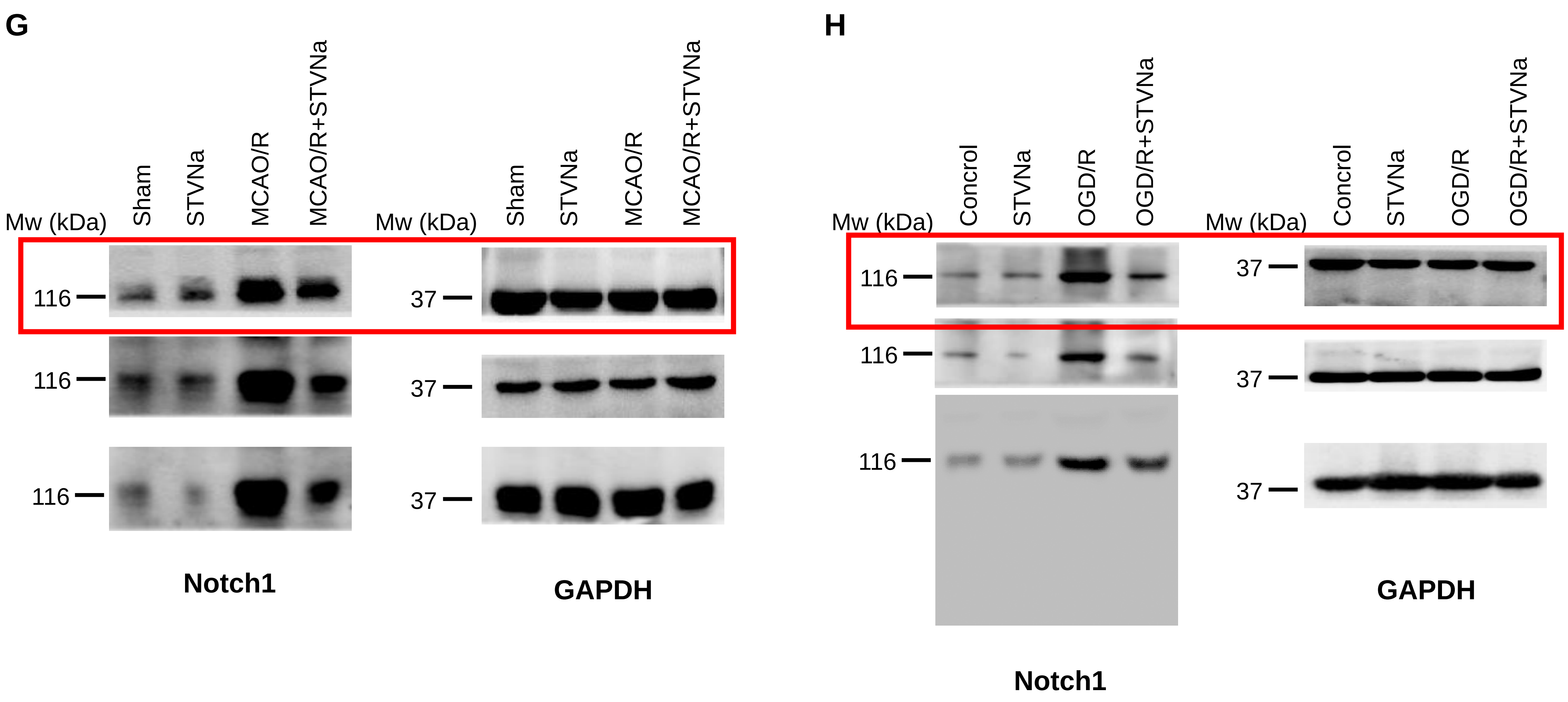
G
Mw (kDa)
Sham
STVNa
MCAO/R
MCAO/R+STVNa
116
37
Notch1
GAPDH
H
Mw (kDa)
Concrol
STVNa
OGD/R
OGD/R+STVNa
116
37
Notch1
GAPDH
